# The Effect of Language on Access to Timely COVID-19 Vaccination of Solid Abdominal Organ Transplant Recipients

**DOI:** 10.3389/ti.2023.10888

**Published:** 2023-02-14

**Authors:** Claire M. de Crescenzo, Ya-Wen Chen, David C. Chang, Heidi Yeh

**Affiliations:** Massachusetts General Hospital and Harvard Medical School, Boston, MA, United States

**Keywords:** transplant recipients, COVID-19 vaccination, healthcare access, language, disparities

## Abstract

In dynamic healthcare environments including the COVID-19 pandemic, it is paramount to communicate health recommendations expediently and clearly. Research has shown social determinants of health affect the impact of COVID-19 on abdominal transplant recipients, but there has been less research on the effect of language proficiency. This is a cohort study of time to first COVID-19 vaccination among abdominal organ transplant recipients in an academic medical center in Boston, MA between 18 December 2020, and 15 February 2021. Cox proportional hazards analysis of time to vaccination by preferred language were adjusted for race, age group, insurance, and transplanted organ. Among 3001 patients, 53% were vaccinated during the study period. Language preference other than English was independently associated with delay to vaccination (0.64, *p* = 0.001), on adjusted analysis. In addition, Black, Hispanic and other race patients were less likely to be vaccinated than white patients (0.58, 0.67, 0.68 vs. reference, all *p* < 0.03). Language preference other than English is an independent barrier to solid abdominal organ transplant recipients’ access to timely COVID-19 vaccination. Equity in care should be improved by providing targeted services to minority language speakers.

## Graphical Abstract

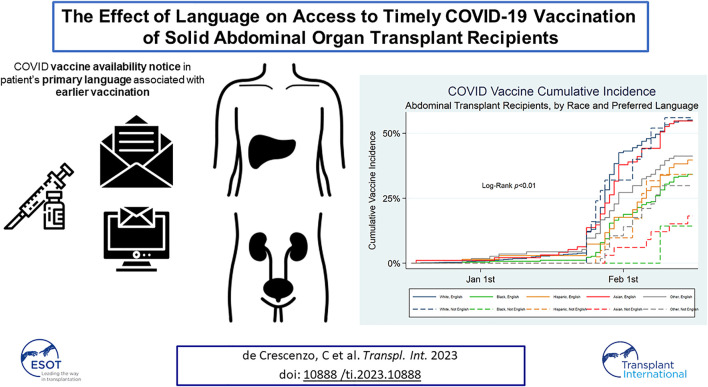

## Introduction

Limited English proficiency (LEP) is increasingly recognized as an independent barrier to timely healthcare access and optimal outcomes (1–3). In the context of the dynamic COVID-19 pandemic, healthcare communication with the public is vital for timely access to testing, treatment, and vaccination. Prior research has established that patients with LEP, even within racial groups, have increased risk of contracting COVID-19, and of needing hospitalization after a positive COVID-19 test (4–6). A rubric has been proposed for improving clinical care of patients with LEP who test positive for COVID (7). However, it can be difficult to determine the contributory effect from various social determinants of health as patients with LEP may also have socioeconomic barriers to accessing timely healthcare.

Solid organ transplant recipients have been shown to have an elevated risk of testing positive for COVID-19, and of morbidity and mortality from the effects of the virus (8,9). Fortunately, these patients often receive long-term follow-up care from their transplant institution and thus are connected to a healthcare institution and hence more likely than the general population to receive targeted healthcare communication. Vaccinations against COVID-19 received U.S. Food and Drug Administration Emergency Use Authorization on 11 December 2020, but doses were limited. Abdominal solid organ recipients were identified early as a priority group to vaccinate. Abdominal organ transplant recipients receiving care at an academic medical center in Boston, MA were notified of their eligibility by email notice from their transplant team at the medical center in English and Spanish on 22nd January 2021, and paper notices were mailed, starting the same day. These vaccine doses were available for administration only *via* the academic medical center, as this was prior to vaccine availability in community-based vaccination centers. This study analyzes factors that affected time to vaccination for solid organ transplant patients upon vaccine eligibility, to assess for a disparity in access to timely vaccination among this immunosuppressed population who has established care with the transplant care team.

## Materials and Methods

### Data Source, Inclusion, and Exclusion Criteria

Abdominal organ (kidney or liver) transplant recipients receiving care at an academic medical center in Boston, MA were examined through the institutional electronic medical record (EMR) in this study. Patients were included who had a liver transplant, kidney transplant, or both organs. Patients were excluded if they did not have contact with the transplant care team in the past year or have missing data.

### Primary Variables

Patients’ preferred language was the primary independent variable and was divided into two groups, English or language other than English. Date of first dose was used as the primary end point. Start day was the date the first participant in the study population received their first vaccination dose, 18th December 2020. In Massachusetts, COVID vaccinations began 15th December 2020 with healthcare workers, then were extended in stages to care facility residents, elderly and those with certain medical conditions including solid organ transplant recipients. Institutional medical interpreters were overloaded with clinical interpreting for the high census of inpatient COVID patients at the time, but the transplant clinic wanted to share this availability with patients as soon as possible. Bilingual clinic staff translated the notice into Spanish as this was the most commonly spoken non-English language among this population. Patients were notified of the availability of COVID-19 vaccine doses and their eligibility *via* dual language email and paper notices sent in both English and Spanish on 22nd January 2021. These vaccine doses were available for administration only *via* the academic medical center, as this was prior to vaccine availability in community-based vaccination centers. Patients were censored on 15th February 2021, which coincided with an institutional pause in vaccination due to decrease in supply.

### Statistical Analysis

Factors associated with prolonged time to first vaccination in days were adjusted by the cox proportional hazard model, including sex, race, age group, insurance, and organ transplanted. Races were grouped into white, Black, Hispanic, Asian or other race. The other group includes patients who identified as other, multiracial and those whose race was unavailable. Patients’ primary insurance was grouped into private, Medicare or Medicaid. Significance levels were all set at *p* < 0.05, two-tailed. Statistical analyses were performed using Stata software, version 15.1 (StataCorp, College Station, TX).

## Results

**FIGURE 1:**
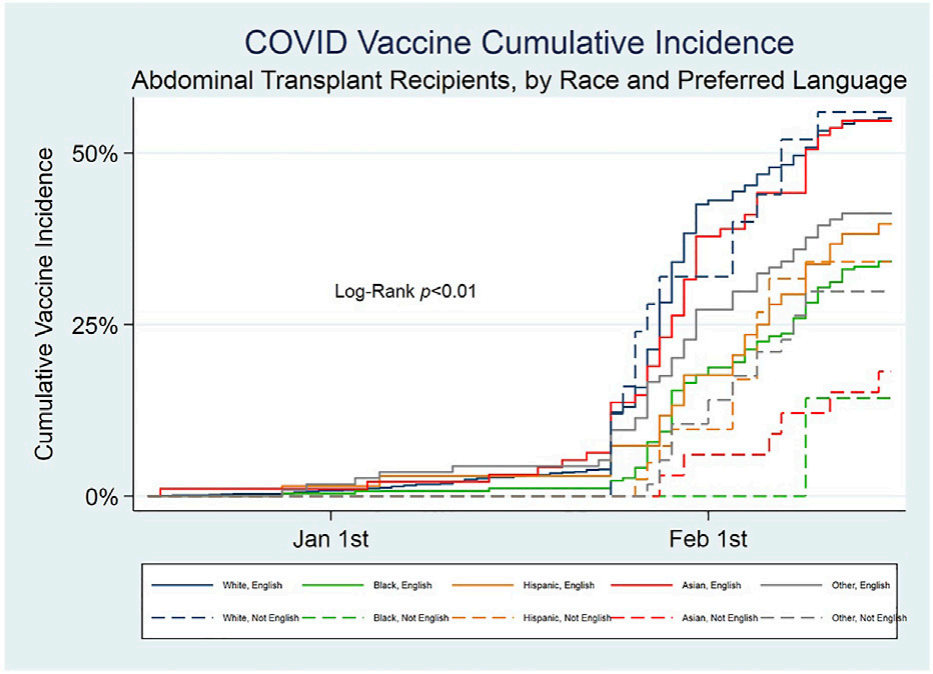
Cumulative incidence of COVID-19 first vaccination among abdominal transplant recipients, by race and preferred language.

There were 3,001 patients that met criteria for inclusion. Nearly 7% of patients had LEP, and the most commonly preferred language other than English among those patients was Spanish (60%) (data not shown). Just over half of the study population (53%) was vaccinated during the study duration. Median date of vaccination was 29th January 2021, 7 days after notification and 42 days after the first patient in the cohort was vaccinated; presumably the few patients vaccinated prior to notification were part of another eligible group such as healthcare providers. The marked increase in vaccination rate then coincides with notifications (Figure 1).

**FIGURE 2:**
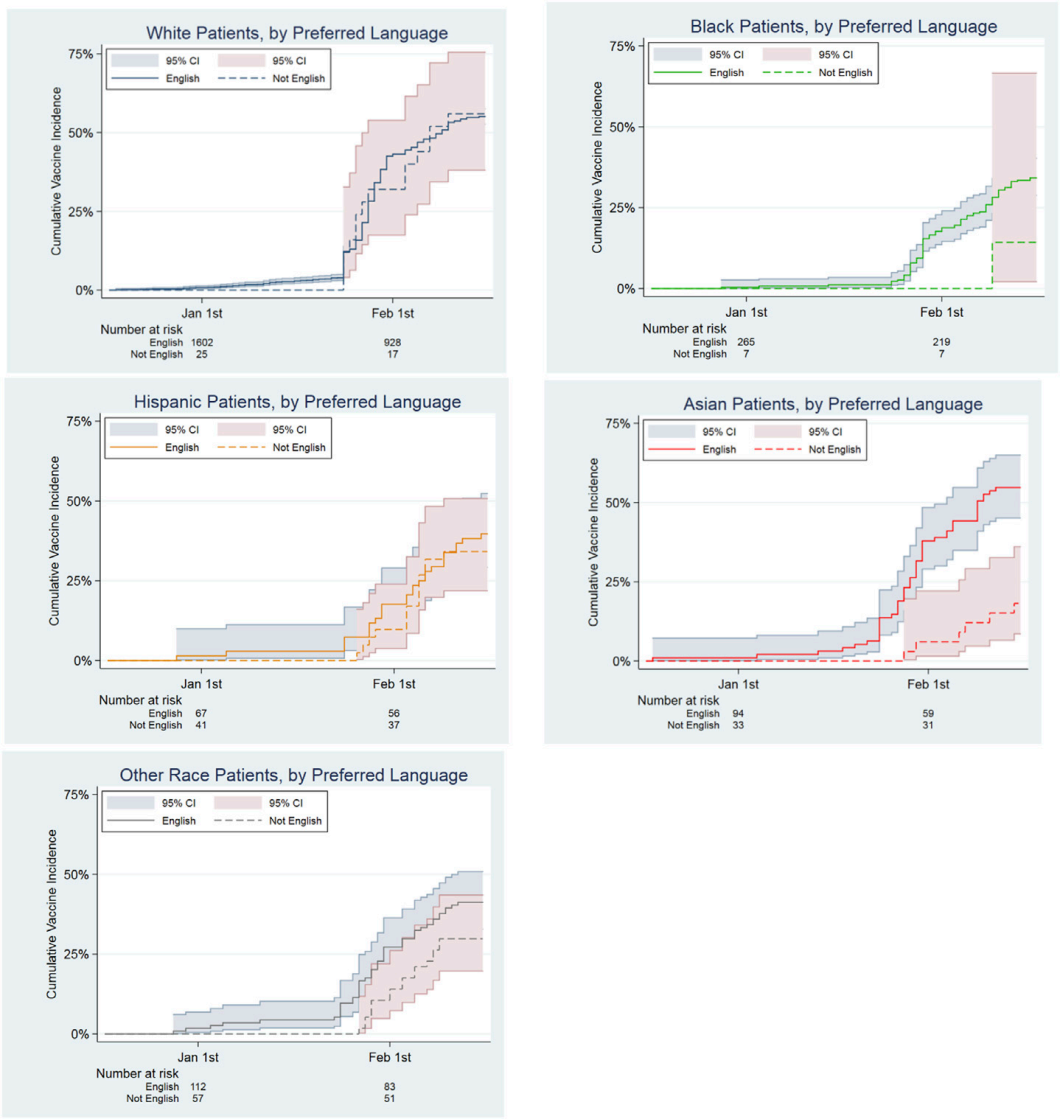
Hazard Ratios of Time to First COVID Vaccination by Race among Abdominal Transplant Recipients, comparing English as Preferred Language to Other Language Preferred.

On unadjusted analysis, patients not yet vaccinated were significantly more likely to have LEP, be younger, have Medicaid insurance, and identify as a race other than white (Table 1). The cox proportional hazard model of vaccination during the study period demonstrated that patients with a preferred language other than English were less likely to be vaccinated (0.64, *p* = 0.001) during the study duration, even after adjustment for race (Table 2). Black, Hispanic and other race patients were less likely to be vaccinated compared to the white group (0.58, 0.67, 0.68; all *p*-value<0.03). When the population is split into groups by race, there is a trend to reduced access among non-white patients with LEP (Figure 2). This is most pronounced in the Black, Asian and other race groups, though the 95% confidence intervals overlap in all but the Asian group. A difference between LEP and EP (English proficient) patients’ likelihood of vaccination was not observed among Hispanic patients.

**TABLE 1:** Unadjusted comparisons of study population.

	*n* = 3,001	Not Vaccinated	Vaccinated	*p*-value
		*n* = 1,496	*n* = 1,505	
Preferred Language not English		130 (8.7%)	63 (4.2%)	<0.001
Female		606 (40.5%)	571 (37.9%)	0.15
Race	White	999 (66.8%)	1,192 (79.2%)	<0.001
	Black	196 (13.1%)	113 (7.5%)	
	Hispanic	73 (4.9%)	42 (2.8%)	
	Asian	88 (5.9%)	79 (5.2%)	
	Other/Multi	140 (9.4%)	79 (5.2%)	
Age	<45	310 (20.7%)	211 (14.0%)	<0.001
	45–64	669 (44.7%)	659 (43.8%)	
	65–74	389 (26.0%)	501 (33.3%)	
	75+	128 (8.6%)	134 (8.9%)	
Insurance	Private	727 (48.6%)	780 (51.8%)	<0.001
	Medicare	667 (44.6%)	667 (44.3%)	
	Medicaid	102 (6.8%)	58 (3.9%)	
Organ	Kidney	1,100 (73.5%)	1,120 (74.4%)	0.81
	Liver	354 (23.7%)	347 (23.1%)	
	Both	42 (2.8%)	38 (2.5%)	

**TABLE 2:** Adjusted regression, likelihood of vaccination during the study time.

	*n* = 3,001	Hazard ratio	*p*-value	95% CI
Preferred Language not English		0.64	0.001	0.5 to 0.8
Female		0.95	0.31	0.9 to 1.1
Race	White	Reference		
	Black	0.58	<0.001	0.5 to 0.7
	Hispanic	0.67	0.02	0.5 to 0.9
	Asian	0.93	0.57	0.7 to 1.2
	Other/Multi	0.68	0.001	0.5 to 0.9
Age	<45	Reference		
	45–64	1.24	0.008	1.1 to 1.4
	65–74	1.51	<0.001	1.3 to 1.8
	75+	1.3	0.02	1.0 to 1.6
Insurance	Private	Reference		
	Medicare	0.87	0.02	0.8 to 1.0
	Medicaid	0.76	0.05	0.6 to 1.0
Organ	Kidney	Reference		
	Liver	0.91	0.15	0.8 to 1.0
	Both	0.87	0.42	0.6 to 1.2

## Discussion

This analysis demonstrates that LEP is an additive barrier to accessing timely COVID vaccination that exacerbates the well-documented disparities related to race. Since at the time of this study vaccines were only available *via* the medical institution, and this population has established care with this institution, there is less heterogeneity than with community-based vaccination and the general community. Undergoing the process of transplantation and follow-up care can provide a degree of healthcare literacy (10) that further decreases the heterogeneity of social determinants of health in this population. While social determinants of health can be difficult to change, our study suggests a potential intervention by the medical community to address the disparities experienced by patients with LEP. Greater than 95% of the LEP Hispanic patients in this study speak Spanish (data not shown), the authors speculate that the reduced disparity among Hispanic abdominal transplant recipients compared to the other non-white races may reflect the impact from the notice being emailed out in Spanish as well as English. This observation suggests the possibility that language support could ameliorate the impact of racial disparity in this population. The potential impact of language support is further suggested in Asian patients, who did not receive language concordant notices. In that case, Asian patients with LEP were less likely to be vaccinated than EP Asian patients and white patients. This suggests that outreach expanded to provide language concordant communication to these patients could improve timely access to care. Though the number of white patients with LEP was small, there was no difference in time to vaccination compared to white patients who speak English, raising concerns that the disparity related to language proficiency predominantly disadvantages transplant patients who are not white. As prior research has argued, there is an onus on the health system to improve access for these patients in an effort to reduce disparities, with potential methods including patient navigators reaching out with medical interpreters, language concordant communication materials, and involving community organizations to develop outreach to these underserved patients (11). The effects of race and language are likely to be even greater in the general population, compared to the post-transplant population in this study. Vaccine availability has improved in the United States, but remains limited in many areas of the world, and these findings suggest attention to the impact of limited proficiency in the local primary language on access to timely vaccination in global communities. These findings regarding disparities in access to timely care merit broader study to determine if there is an exacerbating impact of language proficiency as an independent barrier to access to other important components of healthcare for patients who do not speak the local majority language. In the meantime, efforts should be expanded to provide patients consistent communication in their preferred language.

## Data Availability

The raw data supporting the conclusion of this article will be made available by the authors, without undue reservation.
